# Unique Brain Areas Associated with Abstinence Control Are Damaged in Multiply Detoxified Alcoholics

**DOI:** 10.1016/j.biopsych.2011.04.006

**Published:** 2011-09-15

**Authors:** Theodora Duka, Leanne Trick, Kyriaki Nikolaou, Marcus A. Gray, Matthew J. Kempton, Hugh Williams, Steven C.R. Williams, Hugo D. Critchley, David N. Stephens

**Affiliations:** a School of Psychology, University of Sussex, Brighton, United Kingdom; b Department of Psychiatry, Brighton and Sussex Medical School, University of Sussex, Brighton, United Kingdom; c Experimental Neuropsychology Research Unit, School of Psychology and Psychiatry, Monash University, Monash, Australia; d Sussex Partnership NHS Foundation Trust, Brighton, United Kingdom; e Centre for Neuroimaging Sciences, Institute of Psychiatry, King's College, London, United Kingdom; f Sackler Centre for Consciousness Science, University of Sussex, Brighton, United Kingdom; g Clinical Imaging Science Centre, University of Sussex, Brighton, United Kingdom

**Keywords:** Compulsivity, fMRI, impulsivity, negative patterning, orbitofrontal cortex, reward

## Abstract

**Background:**

The ability to abstain from drinking, despite incentives to imbibe, is essential to recovery from alcoholism.

**Methods:**

We used an incentive conflict task to investigate ability to abstain from responding during presentations of incentive cues. Both alcoholic (*n* = 23) and healthy subjects (*n* = 22) were required to withhold responding during the simultaneous presentation of two visual stimuli in which the individual presentation allowed responding for monetary reward. Brain structures activated during performance of the task were studied using functional magnetic resonance imaging in healthy volunteers (*n* = 8), and changes in gray matter volume were studied in a separate group of patients (*n* = 29) compared with control subjects (*n* = 31) in regions of interest identified on functional magnetic resonance imaging.

**Results:**

Abstinent alcoholic patients were severely impaired on the incentive conflict task. The impairment was greater in patients with experience of several versus a single detoxification. Healthy volunteers, during the same incentive conflict task, showed distinct patterns of brain activation (including gyrus rectus, ventromedial prefrontal cortex, and superior frontal gyrus). Reduction of gray matter volume in ventromedial prefrontal cortex and superior frontal gyrus of patients was more extensive in those with multiple detoxifications.

**Conclusions:**

Performance deficits in alcoholics are associated with withdrawal-induced impairments in prefrontal subfields, which are exacerbated following repeated episodes of detoxification. Detoxification thus compromises functional and structural integrity of prefrontal cortex and may thus impair the ability to control future drinking. Performance in the incentive conflict task is a sensitive biomarker for such deficits.

Recent theories of drug and alcohol abuse (1) emphasize the importance of “bottom-up” incentive processes in initiating drinking and of “top-down” cortical control of behavior in the regulation of drug taking. Drug seeking and taking are often initiated by environmental events (cues) that the addict has learned to associate with drug use (2). Such cues activate incentive mechanisms mediated by brain circuitries, including ventral striatum and orbitofrontal and medial prefrontal cortex (3). Whether incentive exposure results in drug-taking depends on the ability of higher-level monitoring functions to interrupt the incentive process. It is increasingly recognized that the brain areas responsible for such higher-level functions are sensitive to disruption by long-term drug use. Alcohol abuse, in particular, may impair processes that contribute to impulse control (4), so that, faced with alcohol-related cues, alcoholics are motivated to drink, but the means of controlling drinking are impaired. In the alcoholic patient attempting abstinence, this conflict between the desire to take the drug and the requirement to abstain is particularly intense.

Aspects of the interaction between incentive learning and behavioral control are encapsulated in a novel task, the incentive conflict task, a version of the negative patterning task used in cognitive psychology (5,6). In this task, the subject first learns that two independent discrete cues (A+ and B+) signal reward availability, so that they acquire incentive properties. However, in a second phase, while the individual cues continue to signal reward availability, when presented together (AB–) they signal unavailability of reward, or the potential for punishment. Thus, when the cues are presented in combination, there is a mixed message: on one hand, the individual cues prompt reward seeking, but at the same time, the combination signals that reward is unavailable, requiring a reevaluation of the reward contingencies and abstinence from reward seeking.

Such control is thought to be mediated by interactions between prefrontal cortical areas and striatal output systems. Alcohol has direct, long-term effects on prefrontal cortex structure (7) and function (8), and such effects are exacerbated in patients who have undergone multiple detoxifications (MDTx) compared with those with single detoxifications (SDTx) (9). We therefore predicted that alcoholic patients would show a deficit in performance of the incentive conflict task and that the deficit would be exacerbated in MDTx patients. The task might then provide a potential marker for loss of control of drug seeking in addicted patients.

To identify brain areas involved in performance of the incentive conflict task, we used functional magnetic resonance imaging (fMRI) neuroimaging of healthy volunteers in the same task adapted to allow us to discriminate between brain activation related to performance of the incentive conflict task and activation reflecting the switch from simple to more complex images during the introduction of the negative compound stimulus. We predicted that whereas the simple reward-predictive stimuli would engage brain areas associated with signaling reward, introduction of the incentive conflict compound stimulus would additionally engage prefrontal areas necessary for censoring the reward-appropriate response. Because the patients were severely impaired in this task, we were unable to correlate patterns of brain activation with level of performance in this population.

Finally, to test whether those areas identified in the fMRI study as activated during incentive conflict performance were changed in alcoholic patients, we used structural imaging to estimate gray matter volumes in these regions.

## Methods and Materials

### Participants

Incentive conflict performance was tested in 45 participants, 23 diagnosed for alcohol dependence and 22 healthy social alcohol drinkers matched for age, gender, and verbal IQ. Structural MRI was performed on 60 participants, 29 alcohol-dependent, and 31 social drinkers. Patients were diagnosed for alcohol dependence by independent clinicians according to DSM-IV (10) or ICD-10 (11) and were seeking treatment as inpatients or outpatients. All patients had been abstinent for a minimum of 2 weeks at the time of the study and were without any psychotropic medication used during detoxification for at least 72 hours before testing.

In a further study, 14 healthy participants were trained in the incentive conflict task to be included in fMRI. Lack of drug and alcohol abuse was assessed (12,13), and the National Adult Reading Test (14) was evaluated.

All studies were approved by the local ethics committee, and participants gave written informed consent.

Recruitment procedures, sample characteristics, and scanning protocols are provided in Methods and Tables S2 and S3 in Supplement 1.

### Incentive Conflict in Patients

Initial training was followed by a test phase (Figure S1 in Supplement 1). Participants were required to press a computer space bar to obtain monetary reward (10 pence) following presentation of either of two single-element visual stimuli, A+ and B+, each presented 24 times on a computer screen in random sequence. Following each stimulus presentation, subjects rated the likelihood of gaining a reward (1 = *unlikely*, 9 = *likely*; see Supplement 1). The four final presentations of A and B were used to determine “awareness” of the cue–reward relationship. Participants were labeled “aware” if the mean of their expectancy ratings for both A+ and B+ was greater than 5. There were no differences between patients and control subjects in expectancy ratings or in the probability of response during training. All subsequent analyses were performed on data from aware participants (those who had learned the first stage). In the next phase, the compound stimulus (AB–) was introduced, intermixed with presentations of the rewarded single element stimuli (A+ and B+). Pressing the space bar following AB– resulted in loss of 10 pence.

### Functional MRI of the Incentive Conflict Task in Healthy Volunteers

During training, in addition to the rewarded A+ and B+ stimuli, we included two additional stimuli, C– and D–, which resulted in loss of money, enabling us to include in the testing stage a control compound stimulus, CD– with no change of valence from C– or D– alone, and the same outcome as compound AB–. Because the stimuli used for A, B, C, and D were counterbalanced across subjects, by comparing the CD– response with the AB– response, we could isolate brain responses specific to the change in valence from reward predictors A+ or B+ to punished AB–. From 14 healthy participants trained in the incentive conflict task, 10 were selected on the basis of successful acquisition to be included in the fMRI; scanning data from two participants were lost because of technical failures.

Following a training session outside the scanner to establish awareness of stimuli–reward contingencies, a single element presentation phase in the scanner (Part 1, Table S1 in Supplement 1) was used to confirm, in the scanner context, the outcomes associated with the single-element visual stimuli (A+, B+, C–, D–) (48 trials). During the incentive conflict test phase (Part 2; 100 trials), two compound stimuli (AB–, CD–) were introduced, intermixed randomly with presentations of the single-element stimuli. The incentive conflict test phase was followed by a reversal phase (Part 3) consisting of 24 trials, during which the A+ stimulus become A– (presented intermixed with B+, C–, and D–), to control for simple reversal of reward contingencies.

For fMRI analysis, independent statistical models were computed for each part of the task, convolving event onsets with the canonical hemodynamic response function (15). Movement regressors were included as regressors of no interest. Regionally specific condition effects were tested by linear contrasts for each subject at different conditions. The resulting contrast images were submitted to second-level random-effects models, which included all subjects as session variables (16,17).

Effects were tested for significance using T contrasts. To protect against false-positive activations, we report effects meeting a threshold T score of 4.0 and a cluster volume exceeding 72 mm^3^ (*k* = 9 voxels) corresponding to activations equivalent to *p* < .05, corrected. This nonarbitrary voxel cluster size was determined by Monte Carlo simulation (1000 iterations, full width half maximum 3 mm; http://www2.bc.edu/_slotnics/scripts.htm) (18–20) to establish an appropriate voxel contiguity threshold (21), using the same parameters as in our study.

Part 1 contrasted rewarded stimuli (A+, B+) versus nonrewarded stimuli (C–, D–) during single element presentations. Part 2 tested incentive conflict by contrasting the nonrewarded compound stimulus (AB–), made up of rewarded single elements A+ and B+, with CD–, made up of never-rewarded elements C– and D–. To identify activity unique to incentive conflict, we tested whether incentive conflict activated the same regions as the other conditions, namely 1) “reward versus nonreward” taken from training trials; 2) “single versus compound” contrast and 3) “sign difference” contrast taken from the incentive conflict phase, and 4) “reversal” (taken from Part 3. Anatomic masks were created from contrasts of these conditions at an uncorrected threshold of *p* < .001.

### Structural MRI in Patients

Five regions of interest (ROIs; putamen, ventromedial prefrontal cortex, gyrus rectus, superior frontal gyrus, supplementary motor area) associated with incentive conflict performance in the fMRI experiment were created with the Wake Forest University (WFU) Pick Atlas toolbox (22). Nonsmoothed gray matter volumes were extracted from these ROIs for each subject using MarsBaR software (23). Total intracranial volume (TIV) was calculated by summing total gray matter, white matter and CSF volumes. There was no difference between groups in TIV (*F*(2,59) = 1.016, *p* = .368).

ROI data were analyzed for the effect of group (control, SDTx, MDTx) in an analysis of covariance model with TIV as covariate.

### Statistical Analysis of Behavioral Data

Data from A+ and B+ were combined for the analysis, as were C– and D–. Data from the patient study were analyzed using a two-way (2 × 2) mixed analysis of variance (ANOVAs) with stimulus (A+ or B+, AB–) as within-subjects factor, and group (patients, control subjects) as between-subject factor. To examine the detoxification effect, a (2 × 3) mixed ANOVA with group (SDTx, MDTx, and control subjects) as between-subject factor was used. Data from the incentive conflict task in the imaging study were analyzed with a two-way (2 × 2) ANOVA with stimulus (single, compound) and change in reward outcome (yes, no) as within-subject factor. For the reversal part, a repeated-measures ANOVA was performed with stimuli (A–, B+) as within-subject factor.

All analyses were performed using SPSS 16.0 (SPSS, Chicago, Illinois).

## Results

### Performance of Alcoholic Patients in the Incentive Conflict Task

Alcohol-dependent participants did not statistically differ from mild-to-moderate social drinkers with no history of alcohol dependence, in age, gender, and premorbid IQ, nor in past or current use of cannabis or other illicit drugs. Table S2 in Supplement 1 summarizes the general population characteristics of both groups.

#### Incentive Conflict Task

**Figure 1:**
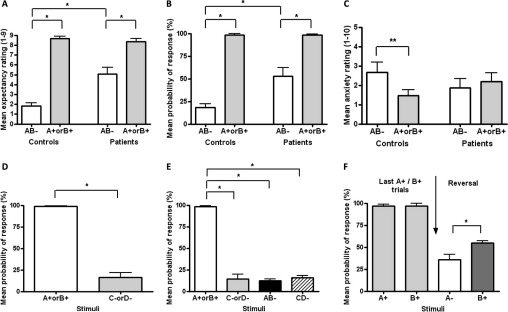
Performance in the incentive conflict task: patients and healthy volunteers. **(A–C)** Performance of alcohol-dependent patients and matched control subjects during different elements of the task: **(A)** expectancy ratings of the response resulting in a 10-pence reward. **(B)** Probability of response. **(C)** Anxiety ratings for the single element stimuli A+ and B+ and the compound stimulus AB–. **(D–F)** Performance of healthy volunteers during imaging when C–, D–, and CD– stimuli were additionally presented, all associated with money loss: **(D)** (reward vs. nonreward) probability of response; **(E)** incentive conflict; and **(F)** reversal. *p < .05; **p < .01, different from comparison group. Data are presented in mean ± SEM.

During training, there was no difference in the number of patients (12 out of 23) compared with control subjects (15 of 22) who became aware of the predictive nature of stimuli A+ and B+ (χ^2^ = 1.20, *p* = .273), allowing us to proceed to the incentive conflict stage with only those subjects who had demonstrated awareness. Following the introduction of the AB– compound, which led to money loss, alcoholic patients incorrectly gave higher reward expectancies than control subjects following AB– [stimulus × group interaction: *F*(1,25) = 21.69, *p* = .001; Figure 1A], although both groups continued to give reward expectancy ratings for A+ or B+ (associated with money gain) higher than ratings for AB–, thus indicating perseveration of contingency awareness [main stimulus effect: *F*(1,25) = 117.90, *p* < .001]. As with expectancy ratings, probability of response was higher overall for A+ and B+ compared to AB– [main effect of stimulus: *F*(1,25) = 130.98, *p* < .001], but patients were markedly more likely than control subjects to make an instrumental response following AB– [stimulus × group interaction *F*(1,25) = 9.74, *p* = .005; Figure 1B]. Whereas control subjects reported higher anxiety ratings for AB– compared with A+ or B+ presentations, indicating its negative emotional valence (Figure 1C), patients gave similar ratings for both stimuli [stimulus × group interaction: *F*(1,25) = 4.39, *p* = .047].

#### Single and Multiple Detoxifications

**Figure 2:**
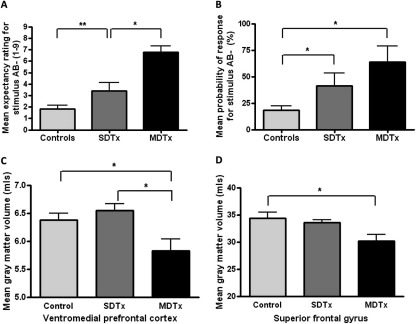
Incentive conflict task in patients, structural magnetic resonance imaging, and number of detoxifications. Expectancy ratings **(A)** and probability of response **(B)** for the AB– compound stimulus in patients divided into those who had undergone a single or no detoxification (≤ 1 SDTx) and those with multiple detoxifications (≥ 2, MDTx), compared with the control subjects. In Panels C and D, gray matter volume was calculated from structural magnetic resonance images for patients (SDTx and MDTx) and control subjects for the ventromedial prefrontal cortex **(C)** and the superior frontal gyrus **(D)** as regions of interest. Gray matter was especially reduced in MDTx patients in these subregions, as was the performance in the incentive conflict task. Note that imaging and behavioral data are from different individuals. The same structures were uniquely activated during performance in the incentive conflict task by healthy volunteers who were able to perform the task. *p < .05; **p < .01, different from comparison group. Data are presented in mean ± SEM.

Following previous practice (9,24), the patient population was divided into two groups consisting of those patients with one or no detoxifications previous to the current one (SDTx, *n* = 15) and those patients with two or more detoxifications (MDTx, *n* = 8). During recruiting, SDTx and MDTx patients were matched for age, gender, verbal IQ, and alcohol consumption before detoxification. In the presence of A+ or B+, expectancy ratings and probability of response were equal for all the groups, and above 8.02 and 97.9%, respectively. However, for AB– presentations, the MDTx group had the highest expectancy ratings of reward and probability of response (i.e., they incorrectly anticipated and responded for reward; Figure 2A and 2B), followed by the SDTx group, followed by the control group [linear stimulus × group interaction, within subjects contrasts: *F*(2,24) = 10.70, *p* < .001 and *F*(2,24) = 6.30, *p* = .007, respectively], indicating that severity of impairment of performance in the incentive conflict task was exacerbated with increased exposure to detoxification.

### Neuroimaging of Incentive Conflict

#### Incentive Conflict Task

For neuroimaging, using healthy volunteers (see Table S3 in Supplement 1 for population characteristics), we adapted the task to include additional stimuli, C– and D–, which never predicted reward. This modification did not affect task acquisition, and during initial training in the scanner, the probability of a response was higher following A+ or B+ (associated with money gain) than C– or D– (associated with money loss; repeated-measures ANOVA showed a significant main effect of stimulus [*F*(1,7) = 224.05, *p* < .001; Figure 1D].

Following introduction of AB– and CD– cues (both associated with money loss), participants were more likely to make a response following A+ or B+ compared with any of the other stimuli (C–, D–, AB–, or CD–; Figure 1E), indicating that they had learned to avoid responding to AB–, as well as to the other negative stimuli. This interpretation was supported by significant main effects of stimulus type [*F*(1,7) = 701.50, *p* < .001] and change [*F*(1,7) = 149.58, *p* < .001], and a significant stimulus type × change interaction [*F*(1,7) = 220.23, *p* < .001].

During the reversal phase (A+ changed from predicting money gain to A– predicting money loss, with B+ remaining unchanged), a significant main stimulus effect on probability of response [*F*(1,7) = 9.00, *p* = .020] reflected that response probability was now lower for A-than for B+ (Figure 1F).

#### fMRI Data: Neural Activity Associated with Incentive Conflict

As anticipated from previous reports (25–27), the contrast between the individually rewarded (A+ or B+) and punished (C– or D–) conditions, presented before introduction of the compound stimuli, was associated with significantly greater activation within medial orbitofrontal (Figure S2A in Supplement 1) and insular cortices (Figure S2B in Supplement 1). Significant activations to reward versus nonreward predictors were also found in posterior and bilateral middle cingulate gyrus, hippocampus, cerebellum, left superior frontal gyrus, and left precentral gyrus (Table S4 in Supplement 1). No significant deactivations associated with the rewarded, relative to the nonrewarded, stimuli were observed.

**Figure 3:**
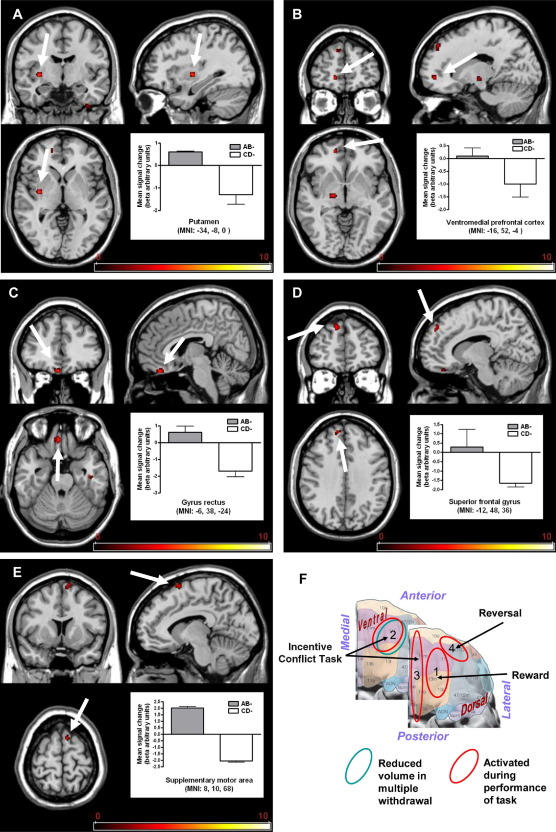
Brain activation to incentive conflict in healthy volunteers. Activity enhancement uniquely associated with incentive conflict (contrast [AB– vs. CD–]): activation within **(A)** the putamen, **(B)** ventromedial prefrontal cortex, **(C)** gyrus rectus, **(D)** superior frontal gyrus, and **(E)** supplementary motor area. Scale represents T statistic. **(F)** Illustration of areas identified in functional magnetic resonance imaging in orbital and ventral aspects of prefrontal cortex as activated during different elements of the task (red circled) and with more severe reductions in gray matter in alcoholic patients associated with multiple detoxifications (blue circles); 1, medial orbitofrontal cortex; 2, ventromedial prefrontal cortex; 3, gyrus rectus; 4, lateral orbitofrontal cortex. Note that the superior frontal gyrus was also activated during incentive conflict and revealed greater gray matter loss in patients with multiple detoxifications. Data are presented in mean ± SEM. MNI, Montreal Neurological Institute.

Brain responses to incentive conflict were revealed through comparison of AB– and CD– (both indicating monetary loss). Our experimental design allowed us to reveal regional activity associated uniquely with the cognitive and motivational processes involved in the task (conflict resolution and regulation of behavioral response) by excluding activity evoked by comparator conditions (presence of reward, presence of compound stimuli irrespective of reward contingencies, or simple reversal) using masking analysis. The highest activity attributable to incentive conflict was observed within the striatum (putamen, Figure 3A). Prefrontal regions were also engaged, notably the ventromedial prefrontal cortex (Figure 3B), gyrus rectus within medial orbitofrontal cortex (Figure 3C), and the superior frontal gyrus (Figure 3D). Consistent with preparation to initiate a response (although the response was withheld) to AB–, the supplementary motor area was activated (Figure 3E). Table S5 in Supplement 1 lists regions with activations associated with processing of the AB– compound.

Our analyses thus identified regions activated by incentive conflict that were different from those activated in comparator conditions, indicating a distinct motivational meaning of the compound stimulus relative to its individual elements. These regions were also distinct from the simple reversal condition, which resulted in significantly greater blood oxygen level–dependent response in the lateral orbitofrontal cortex/lateral frontal pole and medial/superior frontal cortex (Figure S2C and Table S6 in Supplement 1).

Importantly, the three aspects of the task activated different parts of rostral frontal cortex; medial orbitofrontal cortex was activated during the stimulus-reward learning phase, ventromedial prefrontal cortex, gyrus rectus and superior frontal gyrus during the incentive conflict phase, and lateral orbitofrontal cortex during simple reversal (Figure 3F).

### Structural MRI in Alcoholic Patients

Given the impairment seen in alcoholic patients in the task, which were further exacerbated by multiple withdrawals, we asked whether those brain regions identified during imaging the incentive conflict task might be differentially compromised in MDTx patients. Within a more extensive study including 29 alcoholic patients and 31 control subjects (see Table S2 in Supplement 1 for population characteristics), an ROI analysis revealed relative reductions in gray matter volume in alcoholic patients versus control subjects, exacerbated in the MDTx patients, specifically affecting the ventromedial prefrontal cortex [*F*(2,56) = 3.441, *p* = .039; Figure 2C] and superior frontal gyrus [*F*(2,56) = 4.651, *p* = .014; Figure 2D], areas associated with incentive conflict during functional imaging. The effect sizes for the relationships between gray matter volume and number of detoxifications were *r* = –.302 and *r* = –.192 for ventromedial prefrontal cortex and superior frontal gyrus, respectively (Figure S3A and S3B in Supplement 1). These represent small-to-medium effects with gray matter volume and number of detoxifications sharing about 9% of variance in the ventromedial prefrontal cortex and 3.7% in the superior frontal gyrus. Simple between-group contrasts revealed a significantly lower volume in the ventromedial prefrontal cortex for MDTx compared with control subjects (*p* = .011; Cohen's *d* = .624). Similarly, significantly lower volume was found in superior frontal gyrus for MDTx compared to controls (*p* = .005; *d* = .829). This effect size represents well over half a standard deviation difference between control subjects and the other two groups. Another area activated during incentive conflict, the supplementary motor area, showed reduction in gray matter volume in alcoholics [*F*(2,56) = 4.719, *p* = .013], but there was no additional effect of MDTx. No differences were observed in the volumes of putamen [*F*(2,56) = 1.030, *p* = .364] or orbital gyrus rectus [*F*(2,56) = 1.440, *p* = .245; Table 1]. Thus, the detoxification-related deficits in performance of the incentive conflict task in alcoholic patients are attributable to selective detoxification-related damage to a subset of prefrontal areas.

## Discussion

We have demonstrated that alcoholic patients are severely impaired in performing a task that requires them to abstain from responding during presentation of a compound stimulus made up of two cues that individually signal reward availability. The extent of the deficit is remarkable, so that, following further characterization, the task may be useful as a marker of alcoholic dysfunction. However, it remains to be investigated whether individuals with other addictions, and even other impulse or compulsive disorders, may show similar deficits in the task. Furthermore, in accordance with our previous observations (28) that MDTx (withdrawal kindling) increases the severity of the emotional and cognitive impairments seen in alcoholics, in this study patients who had experienced two or more detoxifications showed greater impairment in the task than those with a single experience of detoxification. That both structural and behavioral deficits depended on the number of detoxifications suggests that the deficits are not premorbid but result from the brain damage associated with withdrawal kindling (28–32), perhaps because of increased glutamatergic activity, leading to neuronal toxicity. Nevertheless, from this study we cannot exclude that deficits in performing the incentive conflict task reflect a preexisting condition that also contributed to the development of alcoholism, or tendency to undertake detoxification. For instance, there is some evidence that the number of attempts at detoxification is associated with genetic polymorphisms in the DRD2 gene (33), although in this case it is unclear whether the association simply reflects the higher alcohol consumption associated with the same polymorphisms. However, previous rodent data, in which similar deficits in an incentive conflict task were induced by repeated episodes of ethanol withdrawal (34), are most consistent with the deficit resulting from alcohol consumption and withdrawal. Although MDTx patients are generally likely to have been dependent for longer than SDTx patients, in the patients tested here, there were no differences between the MDTx and SDTx groups in dependency scores, age of starting drinking, or in units of alcohol consumed per week before detoxification (Table S2 in Supplement 1).

Imaging to identify the neuronal substrates involved in the incentive conflict task was performed in healthy control subjects because patients were so seriously impaired in performing the task that we anticipated insufficient data would be obtained from functional imaging in this population. During performance of the incentive conflict task, healthy volunteers showed activation of specific prefrontal areas (including ventromedial prefrontal cortex, orbitofrontal gyrus rectus, and superior frontal gyrus). Related regions, the supplementary motor area and striatum (including putamen), were also engaged. Together, the brain regions activated by AB–presentations are already implicated in the cognitive and emotional processing of reward (dorsal striatum, subregions of orbitofrontal and ventromedial cortex, supplementary motor area) (35–37), as well as with regulatory control over a behavioral response (superior frontal gyrus) (38,39). Activation of gyrus rectus (part of the medial orbitofrontal cortex) is of particular interest because the region is implicated in emotional regulation (25,26). Moreover, its activation in the incentive conflict task parallels its activation by loss of reward through inappropriate performance, which has been interpreted as representing “regret” (40,41).

Importantly, the neural signature of incentive conflict was distinct from patterns of regional activation evoked by simple reversal of reward contingency in which previously reinforced A+ was no longer rewarded (A–), indicating that the AB– signal was not simply due to the change in valence of the constituent stimuli. Furthermore, it differed from presentation of the simple reward-predictive stimuli (A+ or B+), which resulted in a pattern of activation within a different subregion of orbitofrontal cortex (medial orbitofrontal), as well as insular cortex, consistent with previous research examining stimulus-reward response-related learning (27,35,42,43).

The different patterns of activation within orbitofrontal cortex during performance of various components of our task highlight the heterogeneity of function within this region (44). The involvement of the ventromedial prefrontal cortex, an area involved in motivational decision making (45,46), and of superior gyrus, important in behavioral control (38,39), is of particular interest because only MDTx patients showed reliably reduced gray matter volume in this region. Thus, although performance of incentive conflict was associated with activation of several related brain areas, the behavioral deficit in alcoholic patients may reflect damage to only a subset of these regions—in particular, the ventromedial prefrontal cortex and superior frontal gyrus. Activation of ventromedial prefrontal cortex is shared with the gambling task (37), which resembles incentive conflict in requiring decision making. However, incentive conflict additionally involves conflict generated by the contradictory information carried by the AB stimulus, which might be assumed to predict increase of reward (both positive stimuli together) but actually informs of absence of reward.

This study has some limitations. There was no imaging of alcoholics during performance in the incentive conflict task, so we cannot be certain whether their impaired performance was accompanied by failure to activate appropriate brain regions or whether performance was impaired despite their activation. Another limitation is the lack of behavioral data in patients with the single stimuli predicting no reward (C– and D–). Such an inclusion would have clarified whether patients have a general deficit in learning under conditions of confusion between single and compound stimulus presentation.

Together, our findings indicate that structural and functional changes in prefrontal cortex occurring as a direct consequence of drug (47) and/or its withdrawal (30) may impair incentive conflict resolution and thus contribute to behavioral inflexibility and persistence of drug taking despite negative consequences.

The behavioral responses within alcoholic patients reported here reveal specific deficits reflecting conflict resolution associated with an inability to learn to avoid negative outcomes that are important not only in control of drinking but also in daily living. Our findings point to the involvement of specific brain areas in performing these functions in healthy subjects and provide evidence of damage to areas of the brain in alcoholic patients associated with these deficits. We propose that the incentive conflict task may provide a potential marker for loss of ability to abstain from drug-seeking in addicted patients, a type of compulsive behavior.

## Figures and Tables

**Table 1:** Structural Magnetic Resonance Imaging in Alcoholic Patients with Various Numbers of Detoxifications

Variable	Controls (*n* = 31)	SDTx (*n* = 17)	MDTx (*n* = 12)
Putamen	5.28 ± .15	5.34 ± .21	4.91 ± .25
Ventromedial Prefrontal Cortex	6.54 ± .11	6.39 ± .16	5.97 ± .18⁎
Gyrus Rectus	5.02 ± .08	4.96 ± .11	4.68 ± .13
Superior Frontal Gyrus	34.66 ± .66	32.76 ± .96	30.98 ± 1.07⁎
Supplementary Motor Area	12.68 ± .24	11.87 ± .33⁎	11.39 ± .39⁎

Volume of gray matter (mL; mean ± SEM) in each region of interest by group. MDTx, patients with multiple detoxifications; SDTx, patients with single or no detoxification.

⁎ *p* < .05 compared with controls, post hoc independent *t* tests.

## Footnotes

Supplementary material cited in this article is available online.
