# Supplementary material for: Unique Brain Areas Associated with Abstinence Control Are Damaged in Multiply Detoxified Alcoholics
Source: Biol Psychiatry. 2011 Sep 15;70(6):545–52. doi: 10.1016/j.biopsych.2011.04.006 (PMC3165202; doi:10.1016/j.biopsych.2011.04.006)
Supplement: Supplement 1 [file mmc1.pdf]

Supplemental Information

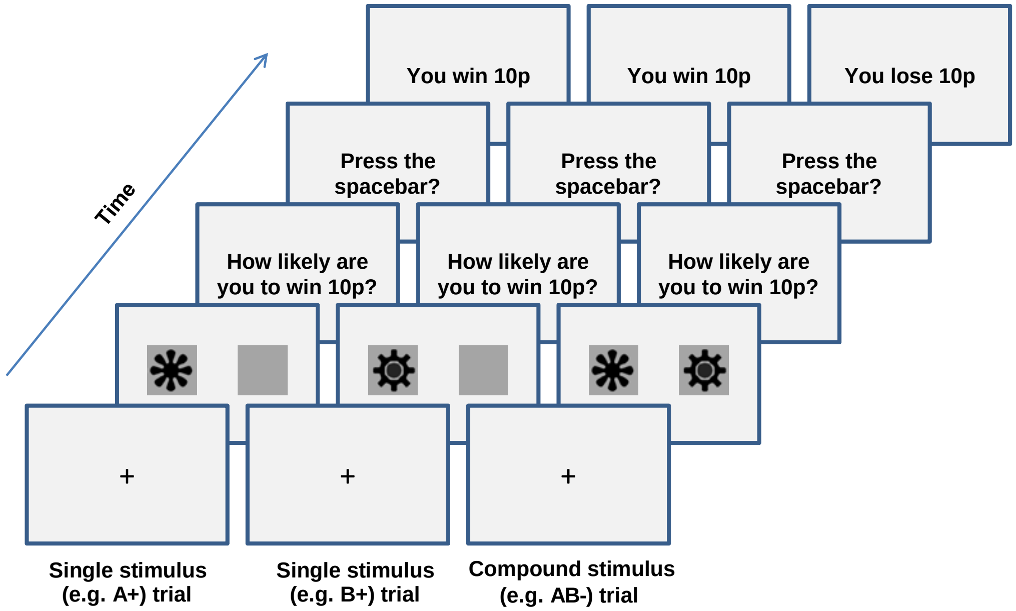

Figure S1. Incentive conflict test procedure. Sequence of task during training (only single stimulus presentation), and at testing when compound stimulus trials were introduced intermixed with single stimulus trials. Each trial started with the fixation cross followed by the visual stimulus presented for 3 seconds, followed by an expectancy question “How likely are you to win 10 pence?”, which was answered by pressing a key between 1 and 9 (1 = unlikely, 5 = don’t know and 9 = likely). Then the prompt “Press spacebar now?” was presented for a maximum of 3 seconds. Pressing the spacebar led to the outcome (displayed for 2 seconds) associated with the stimulus. If the spacebar was not pressed during the prompt nothing happened, and the sequence proceeded to the next trial. During test, presentations of the compound stimulus (AB-), associated with loss of money (incentive conflict), were introduced, intermixed with presentations of the single element stimuli (A+, B+) which continued to be rewarded. The incentive conflict task in the scanner differed from the one used with patients in that two additional stimuli were added (C- and D-) at training, which predicted money losses and the compound stimulus (CD-) at test, which also predicted money loss, in order to serve as controls for the interpretation of imaging data.

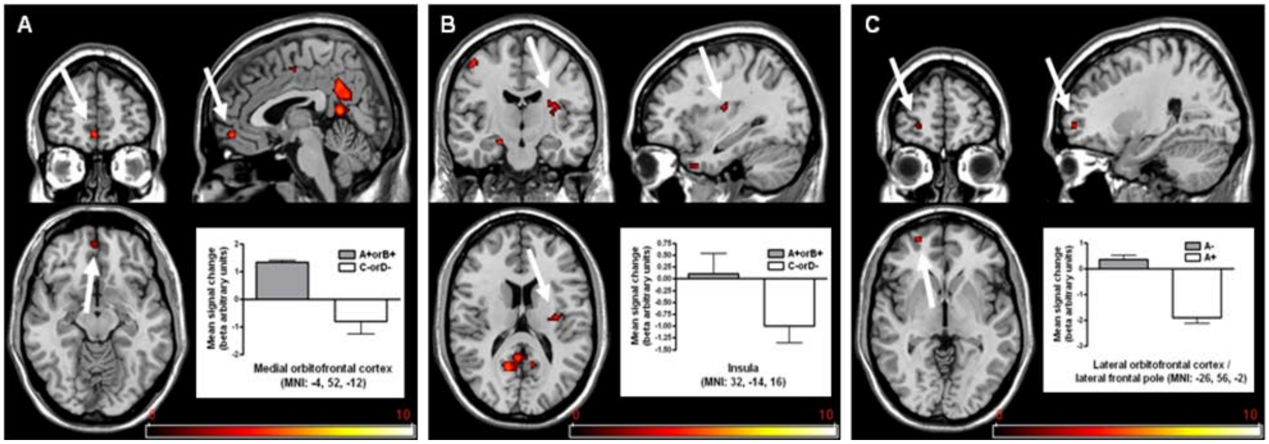

Figure S2. Brain activation to reward and reversal. Activity enhancement within medial orbitofrontal cortex **(A)**, and insula **(B)** associated with reward [A+ or B+ versus C- or D-] and within lateral orbitofrontal gyrus/frontal pole **(C)** associated with reversal [A- versus A+]. Scale represents *T* statistic. Contrasts were used as control conditions for masking brain activations during incentive conflict performance to reveal areas activated which are not associated to learning about reward outcomes (reward versus non reward) or to reversal. Data are presented in mean ± SEM.

A

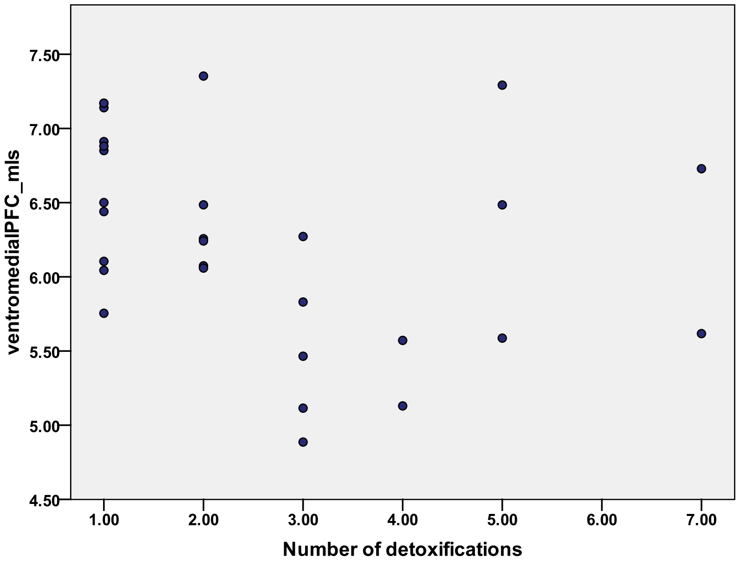

B

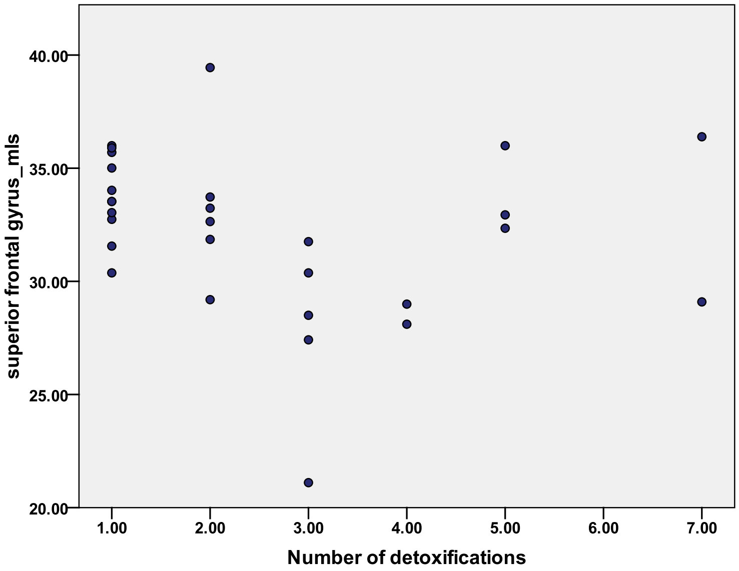

Figure S3. Structural MRI and number of detoxifications. Scattergrams showing the relationship between gray matter volumes and number of detoxifications for the ventromedial prefrontal cortex (r = -0.302; p = 0.111) and the superior frontal gyrus (r = -0.192; p = 0.319).

Table S1. Test procedure in the scanner: Stimulus presentation, number of trials per stimulus and total trials at different phases of performance. Each part was completed before proceeding to the next part. Trials within each part were randomly allocated to each of the stimuli, except that, in Part 2, during the last 24 trials, only the stimuli A+, B+, C-,and D- were presented to allow evaluation of the reversal for stimulus A (A→ A-) during Part 3.

Parts in the procedure	Stimuli	Trials/stimulus	Total trials	Behavioral components
Part 1 (training)	A+	16	48	Single element presentation (reward vs non-reward)
	B+	16		
	C-	8		
	D-	8		
Part 2 (test)	A+	24	100	Incentive conflict
	B+	24		
	C-	4		
	D-	4		
	AB-	32		
	CD-	12		
Part 3 (test)	A-	8	24	Reversal
	B+	8		
	C-	4		
	D-	4		

Table S2. Population characteristics for alcoholic patients and social drinkers groups participating in the incentive conflict task, or in the structural MRI.

Variable	Incentive Conflict Task				Structural MRI			
	**SDTx (*n* = 15)**	**MDTx (*n* = 8)**	**Controls (*n* = 22)**		**SDTx (*n* = 17)**	**MDTx (*n* = 12)**	**Controls (*n* = 31)**	
Age (years)	39.3 ± 11.5	42.8 ± 9.8	40.7 ± 12.2	$F(2,42) = .236$, $p = .791$	37.6 ± 9.6	44.4 ± 9.9	40.2 ± 8.7	$F(2,59) = 1.793$, $p = .176$
Gender	11M, 4F	4M, 4F	15M, 7F	$\chi^2 = .044$, $p = .833$	11M, 6F	7M, 5F	16M, 15F	$\chi^2(2) = .783$, $p = .676$
Full scale IQ	106.1 ± 11.0	100.7 ± 11.5	108.9 ± 8.7	$F(2,42) = 1.962$, $p = .153$	101.9 ± 7.1	106.3 ± 6.3	106.7 ± 7.3	$F(2,59) = 2.619$, $p = .082$
Medical detoxifications*	1.1 ± 0.3	4.0 ± 2.1	N/A	$t(21) = -5.521$, $p < .001$	1.3 ± 0.5	4.3 ± 1.5	N/A	$t(12.6) = -6.645$, $p < .001$
Severity of dependence#	31.3 ± 13.1	40.7 ± 7.4	N/A	$t(21) = -1.865$, $p = .076$	33.1 ± 14.3	46.8 ± 6.4	N/A	$t(23.7) = -3.475$, $p = .002$
Age started drinking	14.1 ± 2.9	16.5 ± 2.5	15.0 ± 1.6	$F(2,42) = 2.948$, $p = .063$	16.5 ± 5.7	17.3 ± 5.9	15.6 ± 2.4	$F(2,59) = .718$, $p = .492$
Alcohol units per week*#	181.9 ± 69.7	182.9 ± 96.7	24.0 ± 31.5	$F(2,42) = 34.301$, $p < .001$	273.5 ± 279.1	336.4 ± 243.6	14.7 ± 14.8	$F(2,59) = 18.592$, $p < .001$
Cigarettes/day	24.3 ± 16.2	28.4 ± 18.8	9.17 ± 6.6	$F(2,25) = 2.848$, $p = .077$	24.6 ± 18.1	23.6 ± 8.2	10.8 ± 4.0	$F(2,27) = 1.192$, $p = .169$
BDI#	12.8 ± 7.0	11.9 ± 9.2	8.90 ± 8.9	$F(2,42) = 1.078$, $p = .350$	18.8 ± 11.3	13.7 ± 11.6	6.4 ± 7.6	$F(2,59) = 9.957$, $p < .001$
State anxiety#	37.3 ± 9.2	40.1 ± 12.7	32.3 ± 10.9	$F(2,42) = 1.918$, $p = .160$	38.9 ± 9.9	39.7 ± 14.1	30.2 ± 7.6	$F(2,59) = 6.347$, $p = .003$
Trait anxiety*#	51.0 ± 11.3	44.1 ± 10.0	38.8 ± 13.7	$F(2,42) = 4.343$, $p = .019$	50.6 ± 10.3	46.3 ± 12.1	36.7 ± 9.8	$F(2,59) = 10.817$, $p < .001$

Values given in mean ± SD.
BDI, Beck Depression Inventory; F, female; M, male; MDTx, multiple detoxifications; N/A, not applicable; SDTx, single detoxifications.
*Significant main group effect, $p < .05$ (incentive conflict).
Significant main group effect $p < .05$ (structural MRI).

Table S3. Population characteristics for the healthy volunteers group participating in the functional MRI (n = 8).

**Variable**	
Age (years)	21.6 (± 1.1)
Gender	3M, 5F
Full scale IQ (NART)	117.1 (± 3.5)
Weekly units	7.6 (± 2.1)
Binge drinking score	17.4 (± 3.6)

Values given in mean ± SEM.
F, female; M, male; NART, National Adult Reading Test.

Table S4. Neuroimaging [Part 1, Single element presentation (reward vs non reward)]: Regions that were activated by rewarded stimuli (A+ or B+) in contrast to unrewarded stimuli (C- or D-) (p = .001, uncorrected, k ≥ 10 voxels).

**Region**	**Cluster size (in voxels)**	**Left/ Right**	**T**	**MNI coordinates x,y,z**
Cerebellum	81	L	7.28	-16, -42, -48
Posterior Cingulate Gyrus	490	L	5.99	-6, -52, 10
Hippocampus	17	L	5.24	-22, -14, -18
Medial Orbitofrontal Cortex	38	L	5.18	-4, 52, -12
Superior Frontal Gyrus	13	L	4.9	-16, 32, 52
Cingulate Gyrus	19	R	4.78	14, -6, 44
		(L)	(4.29)	(-8, -10, 48)
Precentral Gyrus	19	L	4.75	-48, -14, 54
Insula	24	R	4.53	32, -14, 16
Middle Temporal Pole	10	R	4.41	32, 14, -40
Middle Temporal Gyrus	14	L	4.35	-48, -64, 22

MNI, Montreal Neurological Institute.

Table S5. Neuroimaging [Part 2, Incentive conflict]: Activated regions from contrast [(AB-) – (CD-)] (p < .001, uncorrected, k ≥ 10 voxels).

Region	Cluster size (in voxels)	Left/Right	T-value	MNI coordinates x,y,z
Putamen	27	L	4.97	-34 -8 0
Ventromedial Prefrontal Cortex	11	L	4.76	-16 52 -4
Gyrus Rectus	25	L	4.59	-6 38 -24
Supplementary Motor Area	23	R	4.25	8 10 68
Inferior Temporal Gyrus	11	R	4.15	42 -16 -26
Superior Frontal Gyrus	30	L	4.00	-12 48 36

MNI, Montreal Neurological Institute.

Table S6. Neuroimaging [Part 3, Reversal]: Activated regions from contrasts [(A-) – (A+)] and [(A+) – (A-)] (p < .001, uncorrected, k ≥ 10 voxels).

Contrast and Region	Cluster size (in voxels)	Left/ Right	T-value	MNI coordinates x,y,z
**(A-) – (A+)**				
Lateral Orbitofrontal Cortex / Lateral Frontal Pole	12	L	4.19	-26, 56, -2
Medial/Superior Frontal	10	L	4.08	-6, 66, 18
**(A+) – (A-)**				
Superior Occipital	22	L	4.54	-22, -66, 28

MNI, Montreal Neurological Institute.

Supplemental Methods

Incentive conflict task in patients

Participants

Twenty-three alcohol-dependent participants recruited from diagnosed alcoholics seeking treatment as inpatients (*n* = 16) or as outpatients (*n* = 7) were compared to 22 healthy social alcohol drinkers recruited from the local community in the central London, Croydon and Brighton areas. Patients' alcohol dependency severity was evaluated by the Severity of Alcohol Dependence Questionnaire (1), their verbal IQ by the National Adult Reading Test (NART; (2)), their depression scores by the Beck Depression Inventory (BDI; (3)) and their anxiety scores by the Spielberger State and Trait Anxiety Inventory (STAI; (4)). Number of detoxifications, age of starting drinking, units per week, number of cigarettes per day and other related information was taken from the medical history records. Detailed sample characteristics are given in Table S2. Patients were abstinent when tested for a minimum of two weeks and had been medically supported during withdrawal with standard detoxification treatments, including administration of chlordiazepoxide and thiamine. All patients had ceased benzodiazepine treatment at least 72 hours prior to testing. A small number of participants (no differences across groups) were taking selective serotonin reuptake inhibitors (SSRIs). The study was approved by the Kings College Hospital NHS Research Ethics Committee (for participants taking part from the Bethlem Royal Hospital) and the Brighton West Research Ethics Committee (for participants taking part from the Substance Misuse Service in Brighton). The patient population was divided into two groups using information obtained from the medical notes regarding medically supervised

detoxifications, defined as periods of abstinence under medical supervision clearly described in the medical records. Five alcohol dependent patients and 1 control participant completed both the incentive conflict task and also underwent structural MRI.

Incentive conflict task

The incentive conflict task in patients comprised initial training followed by a test phase (Figure S1). Participants were required to press a computer space bar to obtain a small monetary reward (10 pence) following the presentation of two single element visual stimuli, A+ and B+ (see example Figure S1), each presented 24 times on a computer screen in random sequence. Following each stimulus presentation, subjects were asked “How likely are you to gain 10 pence?” and responded on the keyboard using a 1 – 9 scale anchored with 1 = unlikely, 5 = don’t know, 9 = likely. The four final presentations of A and B were used to determine ‘awareness’ of the cue-reward relationship. Participants were labelled as ‘aware’ if the mean of their expectancy ratings for both A+ and B+ was greater than 5. There were no differences between patients and control subjects in expectancy ratings, or in probability of response during training. All subsequent analyses were performed on data from aware participants only (i.e., all those who had successfully learned the first stage of the task). In the next phase, a compound stimulus (AB-) was introduced, intermixed with presentations of the rewarded single element stimuli (A+ and B+). Pressing the space bar following AB- resulted in loss of 10 pence (Figure 1).

Functional MRI of the incentive conflict task in healthy volunteers

Participants

Imaging data from eight healthy participants were included in the imaging analysis. Population characteristics are given in Table S3.

Incentive conflict task

In this version of the task, during training, in addition to the rewarded A+ and B+ stimuli, we included two additional stimuli, C- and D-, that resulted in loss of money, thus enabling us to include a compound stimulus, CD- (to be used as control), in the testing stage, that continued to inform of monetary loss (i.e., no change of valence from C- or D- alone, and the same outcome as compound AB-). Since the stimuli used for A, B, C and D were counterbalanced across subjects, by comparing the CD- response with the AB- response we were able to isolate those brain responses specific to the change in valence from reward predictors A+ or B+ to punished AB-. From the fourteen healthy participants trained in the incentive conflict task, ten were selected on the basis of successful acquisition of the task to be included in the fMRI; scanning data from two participants were not included in the analysis due to technical reasons.

Following a training session outside the scanner to establish awareness of stimuli-reward contingencies, a single element presentation phase in the scanner (Part 1; see Table S1) was used to re-establish, in the scanner context, associations between the four single-element visual stimuli (A+, B+, C-, D-) and the outcome (48 trials). During the incentive conflict test phase (Part 2; 100 trials), the two compound stimuli (AB-, CD-) were introduced, and intermixed randomly with presentations of the single element stimuli (A+, B+, C-, D-). The incentive conflict

test phase was followed by a reversal phase (Part 3) consisting of 24 trials. During the reversal phase, the single element A+ stimulus was reversed to become A- (presented intermixed with B+, C- and D-) to control for effects of a simple reversal of reward contingencies.

For the fMRI analysis three statistical models were computed, one for each part of the task (Table S1).

Part 1, Reward versus non-reward: At first level, modelled conditions included A+ or B+ and C- or D-. Subsequently at a second level, two T-contrasts [(A+ or B+) – (C- or D-)] and the reverse were computed to test for differences in activation between rewarded and non-rewarded conditions.

Part 2, Incentive conflict: To ensure that participants had acquired the new reward contingencies (i.e., learnt not to respond when previously rewarding stimuli A or B were presented in compound), only trials that occurred after a change in responding were analyzed.

Modeled conditions at first level included A+ or B+, C- or D-, AB-, and CD- onsets. These contrast images were included as cells in a one factor ANOVA. Subsequently the T contrast of interest [(AB-) – (CD-)] was computed. To identify activity unique to incentive conflict (i.e., the [(AB-) – (CD-)] contrast), we tested whether incentive conflict shared the same regions activated by the other conditions, namely a) “reward” [the (A+ or B+) – (C- or D-) contrast taken from trials within training (reward versus non-reward)]; b) “single versus compound”, [the [(AB-) + (CD-)] - [(A+ or B+) + (C- or D-)] contrast taken from the incentive conflict phase]; c) “sign difference” [the [(C- or D-) – (A+ or B+)] contrast also taken from the incentive conflict phase]

and d) “reversal” [the [(A-) – (A+)] contrast taken from the reversal phase]. Anatomical masks were created from contrasts of these conditions at an uncorrected threshold of $p < 0.001$.

Part 3, Reversal: For each subject, a model was computed that included stimuli A-, B+, combined C- and D- from part 3, and also stimulus A+ (from the last trials of part 2) as conditions. The resulting contrast images were entered as cells in a one factor ANOVA with 4 levels. Subsequently, two T-contrasts of interest were computed: (A-) – (A+) and its reverse.

fMRI methods

fMRI was performed on a 1.5-Tesla Siemens MAGNETOM Avanto MRI scanner (Siemens, Erlangen, Germany) (quadrature birdcage transceiver headcoil) at the Clinical Imaging Science Centre, University of Sussex. Echo planar images sensitive to T^2 blood oxygenation level-dependent (BOLD) contrast were acquired, covering the entire head (36 slices, 3 mm isotropic voxels, TR 3300 ms, TE 50 ms, 64 x 64 matrix). Slices were angled -30° in the anteroposterior axis to reduce susceptibility-induced BOLD sensitivity losses in orbitofrontal regions (5, 6). Functional data were acquired in one continuous session (315 volumes per subject, discarding the initial 4 volumes to ensure steady state B0 magnetization).

Anatomical images of each subject’s brain were collected using a T1-weighted magnetization-prepared rapid acquisition gradient echo (MP-RAGE) sequence (56 X 256 matrix, 0.9 mm isotropic voxels).

The fMRI data were preprocessed and statistically analyzed using SPM5 (Wellcome Trust Centre for Neuroimaging, University College London, UK) and MATLAB 7 (The MathWorks, Inc., Natick, MA). Functional images were realigned and motion corrected, spatially normalized to standard

MNI (Montreal Neurological Institute) space (7); re-sampled to 2 mm isotropic voxels and smoothed (8 mm full-width half-maximum Gaussian kernel).

Structural MRI in patients

Participants

Sixty participants were included in the analysis of whom 29 were alcohol dependent (see Table S2). Social drinkers were recruited from the local community in the central London area. All evaluation procedures and inclusion criteria were the same as with participants (alcoholic patients and social drinkers) who were examined in the incentive conflict task. Five alcohol dependent patients and 1 control participant completed both the incentive conflict task and also underwent structural MRI. The study was approved by the Kings College Hospital NHS Research Ethics Committee.

Structural MRI methods

High resolution T1-weighted structural images from each participant were segmented into gray matter, white matter and cerebrospinal fluid (CSF) volumes using unified segmentation in SPM5. Images were normalized into standard stereotactic space and modulated by the deformation parameters such that each voxel retained local volume information of the original scan.

The images were acquired using a 3T General Electric Signa System MRI scanner at the Maudsley Hospital, London. Images were acquired in the coronal plane using a T1-weighted, three-dimensional spoiled gradient recalled echo protocol (echo time = 2.8 ms, repetition time = 7.0 ms, inversion time= 450 ms, flip angle = 20°, slice thickness = 1.1 mm, in plane resolution = 1.09 x 1.09 mm, number of excitations = 1).

All images were checked manually for gross structural abnormalities before analysis, and flipped into the axial plane. Analysis was performed using voxel-based morphometry (VBM) with unified segmentation in SPM5 (www.fil.ion.ucl.ac.uk/spm/software/spm5) (8). Unified segmentation performs image registration, magnetic resonance imaging bias field correction and tissue segmentation in one generative model. Information regarding regional volume was entered into the segmented data using modulation by the deformation parameters required to normalize the images.

Supplemental References

1. Stockwell T, Murphy D, Hodgson R (1983): The severity of alcohol dependence questionnaire: its use, reliability and validity. *Br J Addict* 78:145-155.
2. Nelson HE, O'Connell A (1978): Dementia: the estimation of premorbid intelligence levels using the New Adult Reading Test. *Cortex* 14:234-244.
3. Beck AT, Steer RA, Brown GK (1996): *BDI-II Manual*. San Antonio, TX: The Psychological Corporation.
4. Spielberger CD, Gorusch RL, Lushene RE (1970): *Manual for the State-Trait Anxiety Inventory*. Palo Alto, CA: Consulting Psychologists Press.
5. Deichmann R, Gottfried JA, Hutton C, Turner R (2003): Optimized EPI for fMRI studies of the orbitofrontal cortex. *Neuroimage* 19:430-441.
6. Weiskopf N, Hutton C, Josephs O, Deichmann R (2006): Optimal EPI parameters for reduction of susceptibility-induced BOLD sensitivity losses: a whole-brain analysis at 3 T and 1.5 T. *Neuroimage* 33:493-504.
7. Ashburner J, Friston KJ (1999): Nonlinear spatial normalization using basis functions. *Hum Brain Mapp* 7:254-266.
8. Ashburner J, Friston KJ (2005): Unified segmentation. *Neuroimage* 26:839-851.
